# Pea and soy fortified with leucine stimulates muscle protein synthesis comparable to whey in a murine ageing model

**DOI:** 10.1007/s00394-024-03506-8

**Published:** 2024-11-21

**Authors:** Francina J. Dijk, Miriam van Dijk, Justin Roberts, Ardy van Helvoort, Matthew J.W. Furber

**Affiliations:** 1 Danone Global Research & Innovation Center B.V., Utrecht, The Netherlands; 2 Cambridge Centre for Sport and Exercise Sciences, Anglia Ruskin University, Cambridge, UK; 3 NUTRIM School of Nutrition and Translational Research in Metabolism, Maastricht University Medical Center +, Maastricht, The Netherlands

**Keywords:** Ageing, Leucine, Muscle protein synthesis, Pea protein isolate, Plant-based, Soy protein isolate, Whey protein isolate

## Abstract

**Purpose:**

To meet the global dietary protein demands, a trend towards plant-based protein (PBP) sources to replace animal-derived protein is currently ongoing. However, PBPs may not have the same anabolic capacity to stimulate muscle protein synthesis (MPS) as dairy proteins. For vulnerable populations with specific medical needs, it is especially important to validate the anabolic properties of PBPs. In this study, a blend of pea and soy protein isolate, with or without additional leucine, was compared to whey protein isolate on MPS in aged mice.

**Methods:**

25-Months aged C57BL/6J-mice received an oral gavage with 70 mg of whey protein isolate (W), PS protein isolate (PS; ratio 51:49), PS fortified with 19% leucine (PS + L), or 0.5mL water (F). Mice were subcutaneously injected with puromycin (0.04 µmol/g body weight, t = 30 min) and sacrificed 60 min thereafter. Left *m. tibialis anterior* (TA) was used to analyse MPS by the SUnSET method and mTOR signal transduction proteins. Amino acid concentrations were determined in plasma and right TA. Dried blood spots (DBS) were analysed for postprandial dynamics of amino acids at 10-20-45-60-min.

**Results:**

MPS was significantly increased by W and PS + L (*p* < 0.003), however not by PS. Pathway protein 4EBP1 showed significant increases with W, PS and PS + L to F (*p* < 0.0002). W and PS + L increased plasma and muscle free leucine equally, which was confirmed by DBS.

**Conclusion:**

A PS blend fortified with leucine stimulates MPS comparable to whey protein in this acute murine ageing model. Leucine appears to be the main driver for the anabolic responses observed.

## Introduction

In order to provide the nutritional needs of the expanding global population, a paradigm shift towards establishing ecological viable food systems becomes an imperative [1]. This is particularly significant concerning protein sources, requiring a transition towards greater incorporation of plant-based protein (PBP) while concurrently reducing reliance on animal-derived protein. This transition is not without its challenges, given that animal-derived proteins generally exhibit superior nutritional profiles compared to plant-based alternatives [1, 2]. Some studies have demonstrated that PBP can have less of an anabolic effect compared to animal-derived proteins due to the difference in amino acid composition with a lower essential amino acid content and a lower digestion and absorption [2–4]. High protein quality is especially important for the elderly and patients, because of their increased need for protein to maintain muscle mass and function [5]. It is known that the ability to respond to meal-associated anabolic stimuli, i.e. amino acids, is impaired during ageing [6, 7] and many studies support the use of animal proteins to overcome this anabolic resistance [8]. Leucine plays an essential role in stimulating muscle protein synthesis (MPS) as an anabolic trigger [8–10]. Animal-derived proteins such as whey are especially high in leucine and have been referred to as the ‘gold standard’ protein for elderly to maintain or regain muscle mass and are therefore also used in medical nutritional products for the frail and elderly [11].

When considering the quality and application of PBP in nutrition therapy it is important not to just look at amino acid profiling [12], but also the bioavailability and functionality of the amino acids, product characteristics such as taste, viscosity, and societal benefits such as cost and sustainability.

In the area of human muscle physiology, to our knowledge, soy protein is the best studied PBP. The rate of digestion of soy falls between casein and whey [13, 14], and several studies show that soy is able to produce an anabolic response, although it is shown to be less potent than whey [15, 16]. Unfortunately, most of these studies are conducted in combination with exercise [17], in young-adult animals models [18] or young human subjects [19] and only a few studies include older subjects with higher anabolic resistance [15]. There is potential for soy as a good alternative to whey, and additional factors such as contained phytochemicals should also be considered since these factors might further influence the anabolic response (reviewed in [20]). In addition to soy, pea protein is a potential candidate as a PBP alternative [21]. Pea protein provides well above the recommended requirement of leucine [22] and has a potentially valuable role in combating the loss of skeletal muscle mass and function in older subjects [23]. This leads to the hypothesis that blending different PBP sources could result in a more balanced amino acid profile with reduction of limiting amino acids, which would help to maximise the anabolic response. Furthermore, leucine content could be increased by including additional free leucine to stimulate MPS [3].

The objective of this study was therefore to investigate whether a PBP blend of pea and soy can stimulate MPS to a comparable level as whey protein which is considered the ‘gold-standard’ for MPS, and whether the addition of free leucine to the pea/soy blend can enhance plant-based MPS acute stimulation in aged mice. These aged mice have been well characterized in a previous study [24] and data could be translated to the human population of 75 years or older [25]. In addition, to understand the underlying mechanisms, the mTOR signalling pathway was studied. To further understand the relevance of leucine and the bioavailability of other amino acids, we measured end point plasma and muscle free amino acid concentrations. Additionally, dried blood spot (DBS) samples were taken during the time course of the acute experiment to gain information about the amino acid dynamics.

## Methods

### Animals

52 Male C57BL/6J mice of 25 months of age (Janvier Labs, Saint Berthevin, France) were individually housed to be aligned with previous long-term studies. Food intake and physical activity were analysed [10, 24] in a climate-controlled room (12:12 dark: light cycle from 6 to 18 lights on, 18 − 6 lights off) with a constant room temperature of 21 ± 1 °C and humidity of 57 ± 2%). Housing consisted of Makrolon Type III cages (Techniplast, Buguggiate, Italy) with standard bedding (Lignocel, BK8/15 radiated, Tecnilab, Someren, The Netherlands) and tissues (Manutan, Den Dolder, The Netherlands). Mice had *ad libitum* access to a semi-synthetic nutritionally complete diet (AIN93M, Sniff, Germany) and had free access to tap water. Drop out due to ageing was anticipated at 9% (meaning 5 animals of the 52). Upon arrival the mice were allowed to acclimatize for 2 weeks and were fasted in the morning starting at 6 am directly after the switch to the light phase and continued for 6 h before supplementations (see experimental protocol) started. During acclimatization, 2 animals were taken out as they reached HEP (human end point) due to ageing, and during sections another 2 animals were excluded due to liver tumour development – this is seen before in the aging model and is not related to any experimental procedure. At section days, animals were stratified randomized by body weight and divided over the 4 experimental groups. During the postprandial period, 1–2 droplets of blood obtained via tail cut, was stamped directly on a protein saver card (Whatman, VWR, Amsterdam, The Netherlands) at 10, 20, 45 and 60 min. This study was conducted under an ethical licence of the national competent authority (CCD, Centrale Commissie Dierproeven), including positive advice from an external, independent Animal Ethics Committee (St. DEC consult, Soest, the Netherlands), and all animal procedures were captured in a protocol approved by the Animal Welfare Body, also Following the principles of good laboratory animal care of Wageningen University. By this process securing full compliance the European Directive 2010/63/EU for the use of animals for scientific purposed.

### Experimental protocol

At section day, mice received an oral gavage (end volume 0.5 mL) containing ~ 70 mg whey protein isolate (W, *n* = 13, 86% pure, Arla Foods Ingredients, Viby, Denmark), 70 mg of a blend of 51% pea protein isolate (84% pure, Roquette, Brussels, Belgium) and 49% soy protein isolate (90% pure, Gushen Biological Technology Group Co., Dezhou City, China) (PS, *n* = 15) or a blend of 70 mg of a pea protein isolate (45%), soy protein isolate (43%) and free leucine (12%) (100% pure, S.A. Ajinomoto OmniChem, Louvin-la-Neuve, Belgium) (PS + L, *n* = 15) or water (fasted control = F, *n* = 5); specifications described in Table 1. Animal numbers per group were based on sample-size calculation, using historical data (see section statistical analysis).

Previously, studies showed that the total quantity of proposed supplemented protein was adequate to generate a positive MPS response in aged mice [10, 26]. The amount of total leucine in the fortified blends was based on the ratio used in medical nutrition: 19% of the total protein content [8, 27]. After the protein supplementation, mice were returned to their home cages, where they were permitted free access to water only. MPS was measured with the SUnSET method as previously described by Goodman et al. using puromycin [28]. Thirty min after oral gavage the mice received a subcutaneous injection with 0.04 µmol/gram body weight puromycin (Sigma-Aldrich, Merck, Zwijndrecht, The Netherlands) [28]. After another 30 min, maximal 1 mL of blood was drawn by cardiac puncture under total isoflurane anaesthesia (isoflurane/N_2_O/O_2_) after which cervical dislocation was applied. Whole blood was collected in heparin coated tubes (12.8 µl heparin (Heparine Leo 5000 I.E./mL from a local Pharmacia) and 7.2 µl of PBS). MPS was measured precisely 60 min after the postprandial period. Hind limb muscles were excised, weighted, frozen in liquid nitrogen and stored at -80 °C until further analysis.

### Protein simple Western™ analysis

Left *tibialis anterior* muscles (TA) were used to measure MPS by SUnSET technique and phosphorylated and total protein of mTOR pathway proteins of 4EBP1, p70S6k and mTOR as described previously in detail by Dijk et al. [26]. Traditional western blotting was replaced by Protein Simple Western™ technique using the Wes™ (ProteinSimple, a Bio-Techne brand, San Jose, USA).

### Dried blood spots

Dried Blood Spots (DBS) samples were taken 10, 20, 45 and 60 min after gavage via tail cut and by immediately pressing the blood droplet on the collection area of a protein saver card, as previously described by Dijk et al. [26]. The DBS of 5 random animals per group were analysed for amino acid profiles by the Aquaculture Centre (University of Stirling, Stirling, UK), as described previously [26]. The analysis was performed for sum of Essential Amino Acids (EAA) (EAA = L-forms of histidine, isoleucine, leucine, methionine, lysine, phenylalanine, threonine, tryptophan, and valine), and separately leucine, isoleucine, valine, arginine, methionine and lysine.

### Biochemical measurements

Plasma was obtained by centrifugation at 1300×g for 10 min at 4 °C. Right TA muscle was freeze dried, homogenized in 2% perchloric acid, centrifuged (2000×g for 20 min at 4 °C) and supernatants were used to determine muscle free amino acid concentrations. Plasma and muscle free amino acid concentrations were measured using ultra-fast liquid chromatography (UFLC) [29]. Essential amino acids (EAA) assessed included L-forms of histidine, isoleucine, leucine, methionine, lysine, phenylalanine, threonine, tryptophan, and valine. Branched-chain amino acids (BCAA) assessed included L-forms of leucine, isoleucine, and valine. Non-essential amino acids (NEAA) assessed included L-forms of alanine, asparagine, aspartic acid, glutamine, glutamic acid, glycine, serine, and tyrosine.

### Relative bioavailability factor

DBS results of leucine were used to calculate the relative bioavailability factor according to the formula:

Relative bioavailability factor = µmol/L leucine from DBS / leucine content in gavage.

For calculation of the max. relative bioavailability factor, the relative bioavailability factor with the highest response was used, i.e., 10 min post gavage. To indicate differences in bioavailability factor in timing between the supplemental groups, the relative bioavailability factor was calculated at all DBS sample times during the experiment. DBS data were corrected for fasted leucine values.

### Statistical analysis

Sample size calculations are based on two-sample t-test for mean difference with (un)equal variances. The primary outcome parameter is MPS. Since this was the first study using 6 h fasting, assumptions on the effect size were made based on previous outcomes performed with prolonged fasting. We hypothesized that the effect size of whey supplementation versus fasted control (*n* = 5) is higher for 6 h fasting than after prolonged fasting, resulting in *n* = 15 for W (power of 99%, assuming SD F = 0.27, SD W = 0.69 and mean MPS difference for Whey vs. F = 1.03, Type I error of 2.5% one sided). Further, PS or PS + L supplementation is assumed to be superior to whey, resulting in *n* = 15 (assuming SD W = SD PS = 0.69, mean MPS difference for PS vs. W and for PS + L vs. W = 1.14; Type I error of 1.25% one sided, group size *n* = 15, individual power of 98%). The study was powered for superiority of W vs. F and for superiority of at least on of PS or PS + L vs. W.

MPS data was checked for normal distribution using Shapiro-Wilk Test. All data were expressed as means ± SEM. Statistical analyses were performed using GraphPad Prism version 9.5.0 for Windows (GraphPad Software, San Diego, CA, USA). One-way ANOVA analysis or mixed-effects model analysis followed by SIDAK *post hoc* analysis was used to compare differences between the 4 groups pair-wisely. Statistical significance was defined as SIDAK adjusted *p* < 0.05. In the text below each SIDAK adjusted p-value is just referred to as “p-value”.

## Results

No remarkable differences in mean body weight, liver and individual hind limb muscles wet weights were found within the experiment (data not shown), assuring groups randomization was successful. Nutritional supplementations were analysed for amino acid profiles shown in Table 1.

**Table 1:** Provided protein supplementations and measured amino acid content

	W	PS	PS + L
Raw material and protein content of gavage (mg/0.5 mL)			
Whey (raw material)	80.9		
Pea (raw material)		42.3	37.2
Soy (raw material)		37.9	33.3
Added leucine			8.4
Total protein	69.9	69.6	69.6
Total leucine	7.0	5.5	13.2
Measured L-amino acid concentration (mg/g gavage)			
Isoleucine	6.9	3.7	2.7
Leucine	12.5	7.4	20.6
Valine	6.3	4.1	2.8
**Sum BCAA**	**25.7**	**15.2**	**26.2**
Histidine	1.7	2.3	1.7
Lysine	9.2	5.3	3.9
Methionine	2.1	0.8	0.3
Phenylalanine	3.3	5.0	3.6
Threonine	8.8	3.7	2.6
**Sum EAA**	**50.7**	**32.2**	**38.2**
Alanine	6.6	4.0	2.8
Arginine	2.8	7.8	5.7
Asparagine + Aspartic acid	12.8	11.4	8.3
Glutamine + Glutamic acid	21.8	17.6	13.2
Glycine	1.6	3.5	2.5
Serine	5.7	5.2	3.8
Tyrosine	3.2	3.6	2.5
**Sum NEAA**	**54.6**	**53.0**	**38.8**
**Total AA**	**105.3**	**85.3**	**77.0**

Values represent data from a single measurement; therefore, no SEM is available

### In vivo muscle protein synthesis

**Fig. 1:**
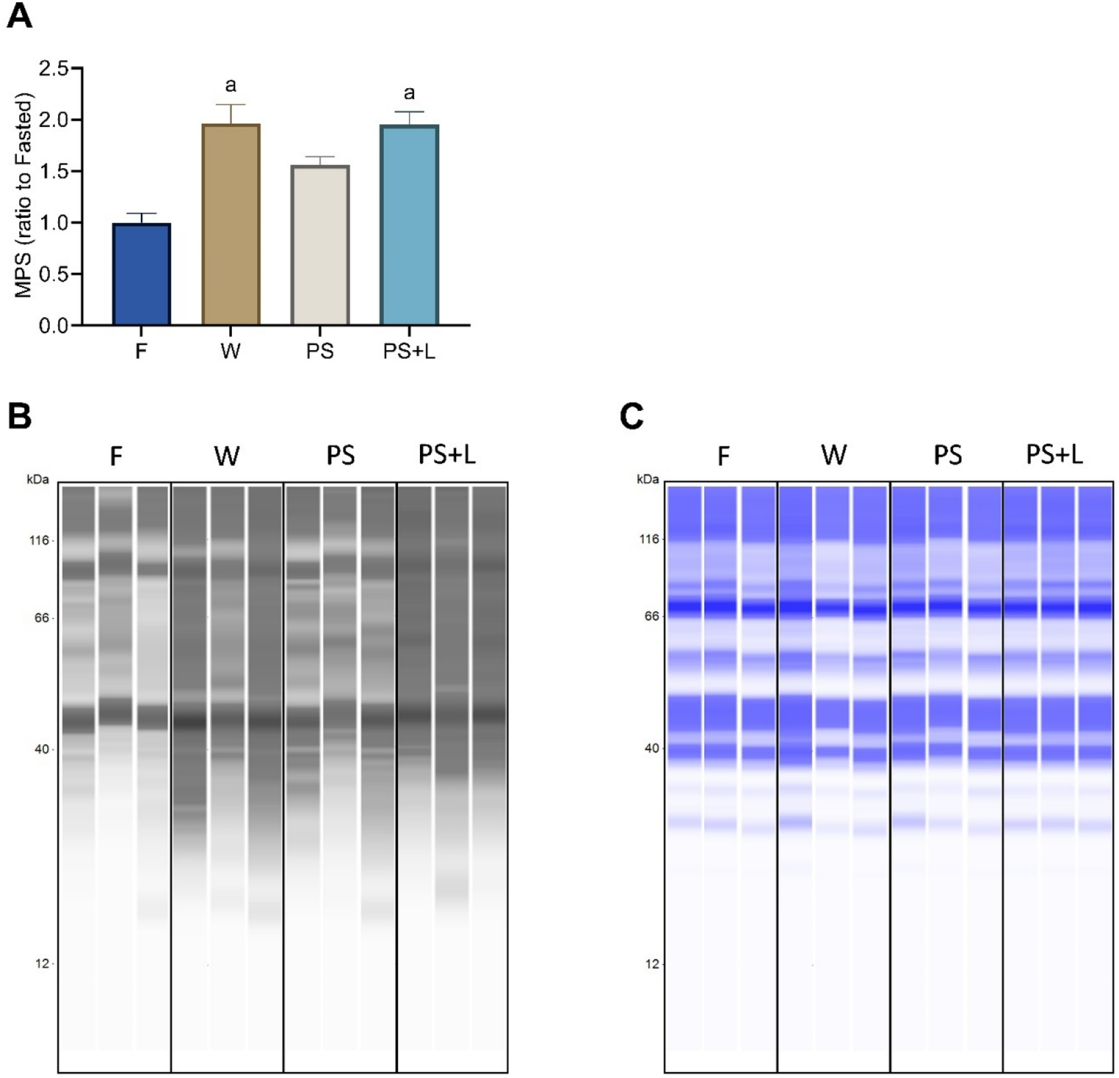
Muscle protein synthesis assessed by the SUnSET method and measured in the TA muscle after 6 h fasting in aged mice after oral gavage with water (F), whey (W), pea and soy (PS) and pea and soy fortified with leucine with equal total protein (PS + L). Values are means ± SEM. (**A**) Muscle protein synthesis in muscle *tibialis anterior*, (**B**) Wes lanes of puromycin incorporation of representative samples; (**C**) Wes lanes of total protein of representative samples. Significant difference to F is represented as ‘a’ (One-way ANOVA with SIDAK *post hoc* analysis, *p* < 0.05)

Oral gavage with W resulted in a 2-fold increase in muscle protein synthesis (MPS) response (*p* = 0.003) compared to F whereas PS did not show a significant increase (*p* = 0.162 vs. F) (Fig. 1A). However, when PS was fortified with leucine, PS + L significantly enhanced MPS (*p* = 0.003 vs. F) and showed a similar observed MPS response as W (*p* > 0.999 W vs. PS + L).

### mTOR signalling proteins

**Fig. 2:**
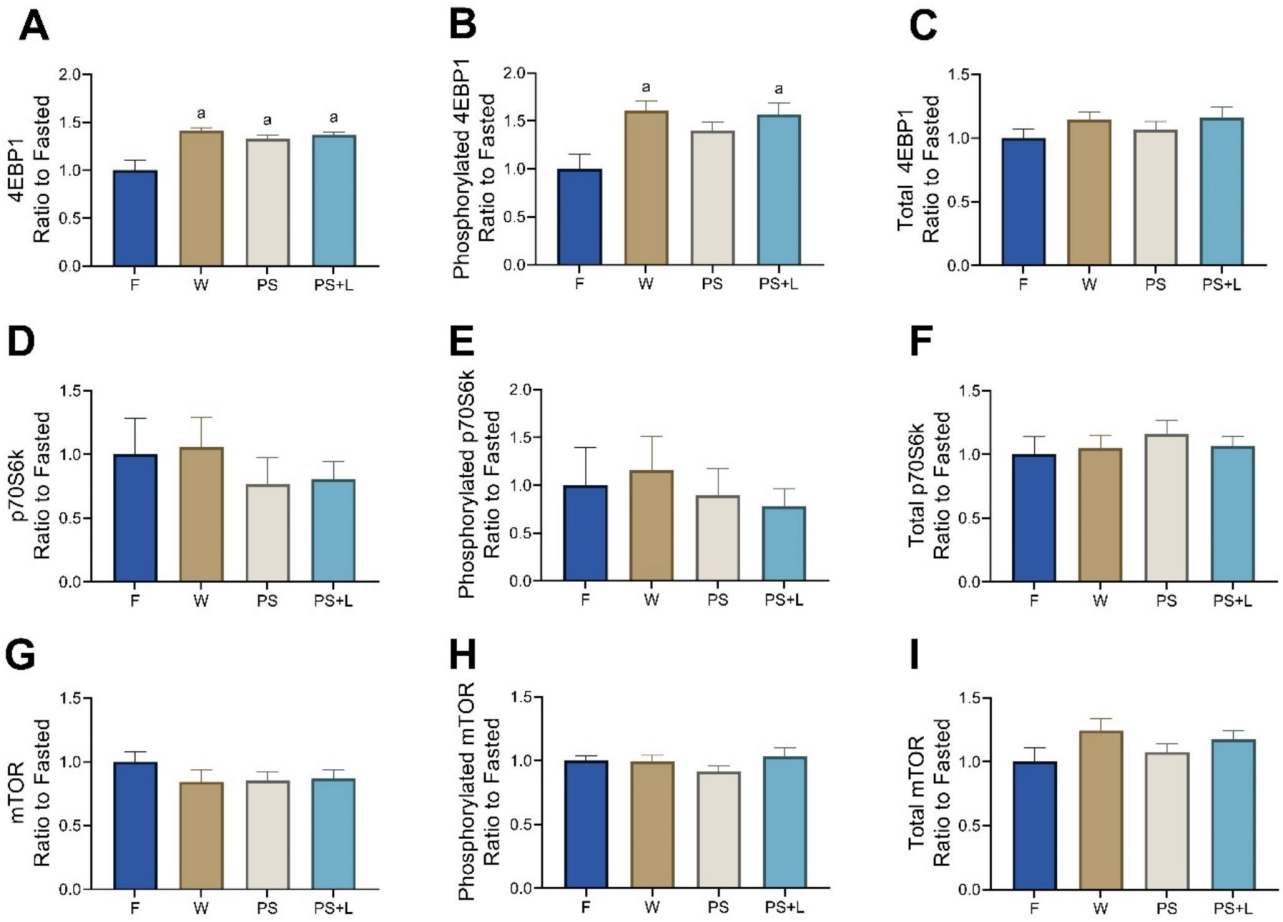
mTOR signalling proteins 4EBP1, p70S6k and mTOR, displaying phosphorylated to total ratios of the TA muscle after oral gavage of water (F), whey (W), pea and soy (PS) and pea and soy fortified with leucine (PS + L). (**A**) 4EBP1 ratio between phosphorylated and total 4EBP1, (**B**) phosphorylated protein of 4EBP1, (**C**) total protein of 4EBP1, (**D**) p70S6k ratio between phosphorylated and total p70S6k, (**E**) phosphorylated protein of p70S6k, (**F**) total protein of p70S6k, (**G**) mTOR ratio between phosphorylated and total mTOR, (**H**) phosphorylated protein of mTOR, (**I**) total protein of mTOR. Values are means ± SEM. Significant difference compared to F is shown as ‘a’ (One-way ANOVA with SIDAK *post hoc* analysis, *p* < 0.05)

Phosphorylated to total ratio of 4EBP1 protein was significantly increased with W, PS and PS + L compared to F (all *p* < 0.0002) (Fig. 2A), whereas phosphorylated 4EBP1 was only significantly increased for W and PS + L (*p* < 0.037 vs. F) (Fig. 2B) and no differences were observed in total 4EBP1 protein (Fig. 2C). Phosphorylated to total p70S6k and mTOR protein did not show any significant changes between conditions (Fig. 2D, G), neither did phosphorylated or total p70S6k or mTOR protein (*p* > 0.05; Fig. 2E, F, H, I).

### Amino acids in dried blood spots

**Fig. 3:**
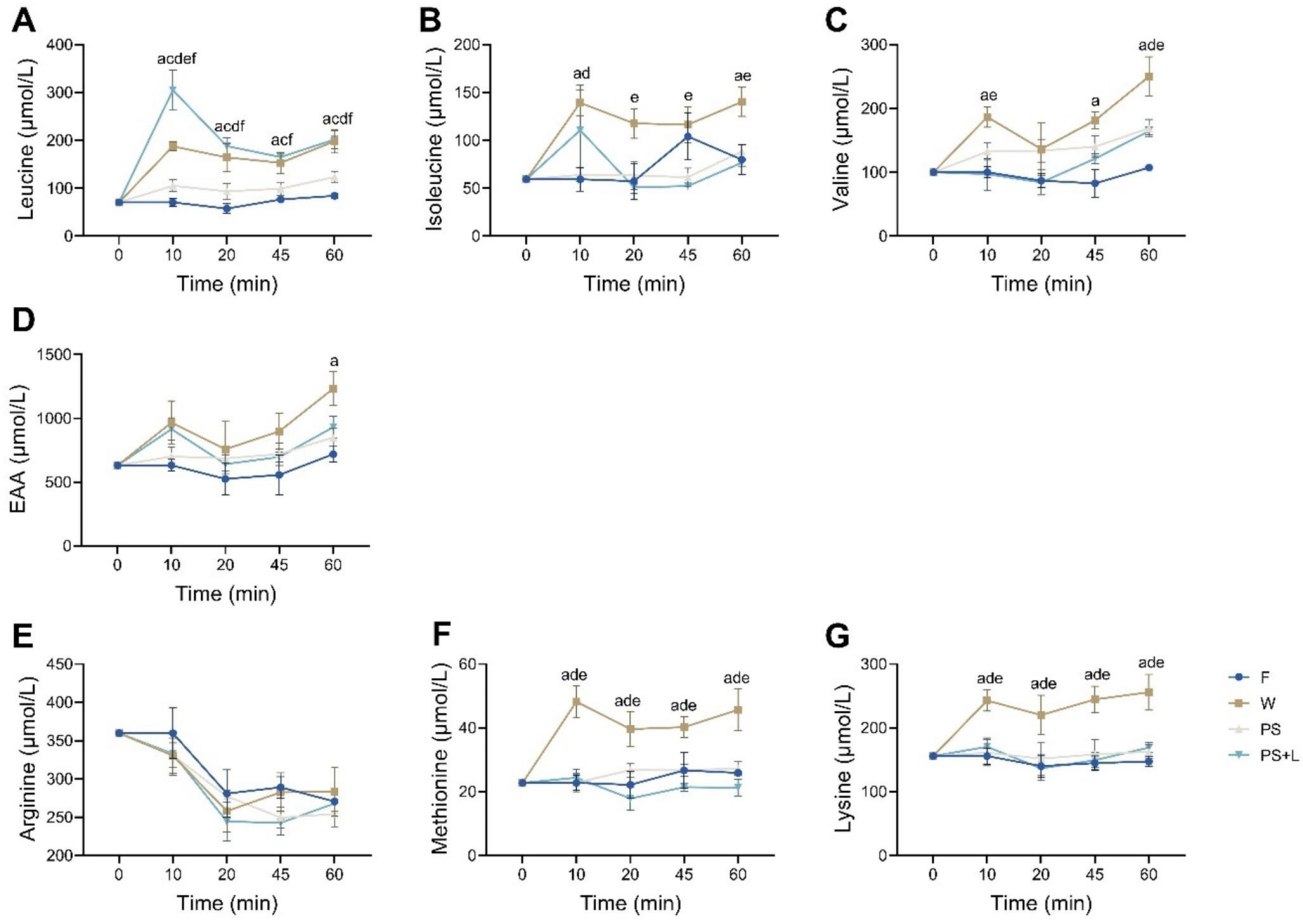
Amino Acids determined from dried blood spot at different time points post supplementation: 10-, 20-, 45- and 60-min. F: fasted, W: whey, PS: pea and soy, PS + L: pea and soy fortified with leucine. **A**) leucine, **B**) isoleucine, **C**) valine, **D**) EAA, **E**) arginine, **F**) methionine and **G**) lysine. Values are means ± SEM. Significances are shown by *a*: F to W, *b*: F to PS, *c*: F to PS + L, *d*: W to PS, *e*: W to PS + L, *f*: PS to PS + L tested with mixed-effects analysis (time curves) with SIDAK *post hoc* analysis, *p* < 0.05

Figure 3 shows the DBS results in a time curve of the amino acid, and Table 2 shows the area under the curve (AUC) of the complete time course. Leucine was significantly increased at all time points after oral gavage with W and PS + L compared to F (*p* < 0.0153) (Fig. 3A). Leucine of PS + L were significant higher compared to W at 10 min (*p* < 0.0001), while PS + L was significant higher compared to PS at all time points (*p* < 0.03). Leucine AUC showed a significant increase compared to F with W and PS + L (*p* < 0.0001), but not with PS (*p* = 0.330) (Table 2). Isoleucine was significantly increased at 10 and 60 min post oral gavage with W vs. F (*p* < 0.005) (Fig. 3B), AUC was significantly increased with W vs. F (*p* = 0.007) (Table 2). Valine was significantly increased with W vs. F at 10, 45 and 60 min post oral gavage (*p* < 0.003) (Fig. 3C), AUC of valine was increased with W vs. F (*p* = 0.0002) (Table 2). EAAs were measured together, and at 60 min W was significantly increased compared to F (*p* = 0.004) (Fig. 3D), AUC of EAA showed no changes between groups (Table 2).

Arginine can act as an anabolic amino acid [30] and was therefore measured, however, the DBS data showed no differences in the experimental groups compared to F (*p* > 0.05; Fig. 3E). Deficiencies of methionine and lysine are often associated with PBP sources. Figure 3F and G showed that these 2 amino acids were not elevated after oral gavage with PBPs. On the contrary, with W supplementation, methionine was significantly increased at all time points after oral gavage compared to F (*p* < 0.014) (Fig. 3F) which also results in a significant increase of the AUC of W (*p* = 0.002) (Table 2). Lysine was significantly increased all time points after oral gavage with W (*p* < 0.007 vs. F) (Fig. 3G), also leading to a significant increase in the AUC with W (*p* = 0.001 vs. F) (Table 2).

**Table 2:** Average area under the curve (AUC) of DBS data of fasted (F) mice, or supplemented with whey (W), pea/soy (PS) or pea/soy fortified with leucine (PS + L)

AUC (µmol/L)	F	W	PS	PS + L
Leucine	4329 ± 263	9676 ± 489^a^	5950 ± 682^d^	11,504 ± 749^ce^
Isoleucine	4349 ± 714	7129 ± 264^a^	3940 ± 467^d^	3915 ± 454^e^
Valine	5677 ± 474	10,415 ± 432^a^	8003 ± 788	6361 ± 599^e^
EAA	35,199 ± 5590	53,299 ± 7095	42,951 ± 4260	44,470 ± 2190
Arginine	18,122 ± 1066	17,736 ± 633	16,860 ± 460	16,268 ± 662
Methionine	1433 ± 133	2463 ± 154^a^	1549 ± 105^d^	1062 ± 228^e^
Lysine	8740 ± 597	13,820 ± 448^a^	9435 ± 1086^d^	9133 ± 680^e^

Values are means ± SEM. Significances are shown by *a*: F to W, *b*: F to PS, *c*: F to PS + L, *d*: W to PS, *e*: W to PS + L, *f*: PS to PS + L tested with one-way ANOVA (AUC) with SIDAK *post hoc* analysis, *p* < 0.05

### Amino acids in plasma and muscle

**Fig. 4:**
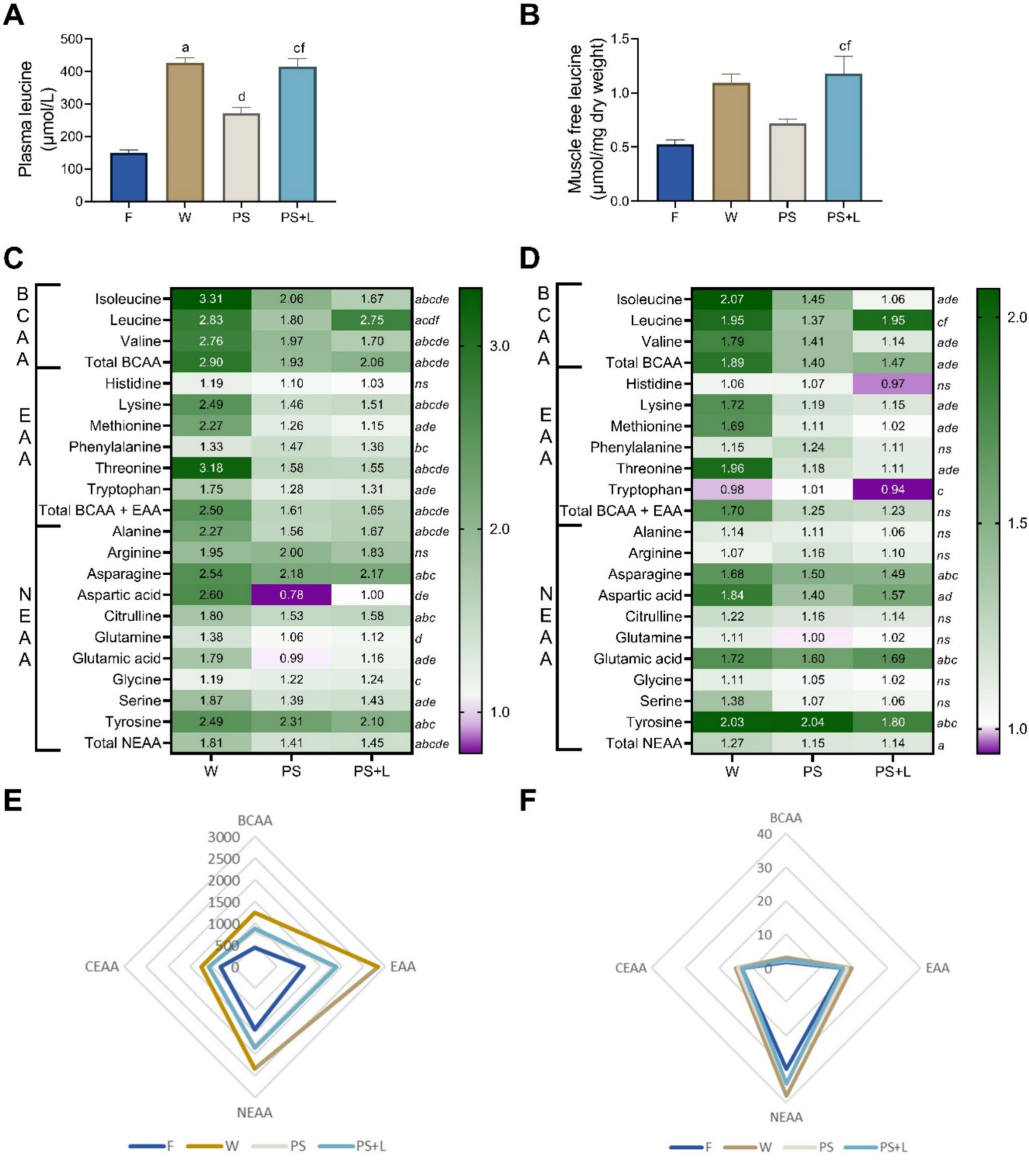
Postprandial amino acid concentrations of plasma and free muscle amino acid concentrations. **A**) Plasma leucine concentrations. **B**) Muscle free leucine concentrations. **C**) Heatmap of plasma amino acid ratios compared to fasted group (F), **D**) Heatmap of muscle free amino acid ratios compared to fasted group. **E**) Radar plot of plasma BCAA, EAA, CEAA and NEAA concentrations. **F**) Radar plot of muscle free BCAA, EAA, CEAA and NEAA concentrations. Values are means ± SEM. Significances in bar graphs and heatmap (*p* < 0.05) are shown by *a*: F to W, *b*: F to PS, *c*: F to PS + L, *d*: W to PS, *e*: W to PS + L and *f*: PS to PS + L, *ns* is not significant. (One-way ANOVA with SIDAK *post hoc* analysis, *p* < 0.05)

Plasma leucine concentrations increased 2.8-fold after W supplementation (*p* < 0.0001) and 1.8-fold after PS compared to F (*p* < 0.062) (Fig. 4A and C; Table 3). Fortifying PS with leucine increased plasma leucine levels 2.8-fold (*p* < 0.0001 vs. F). Further observations were made in the other amino acid levels when comparing the groups: an overview of all amino acid plasma ratios compared with F can be found in the heatmap (Fig. 4C) and absolute values are shown in Table 3. In summary, plasma levels of BCAA, EAA and NEAA were significantly increased vs. F after 60 min independent of which protein supplementation. Compared to W, BCAA, EAA and NEAA levels with PS and PS + L supplementation were significantly lower (Fig. 4C and E).

Free leucine concentrations in muscle were 2.1-fold increased after oral gavage with W (*p* = 0.068), 1.4-fold with PS (*p* = 0.903), and 2.2-fold with PS + L (*p* = 0.025) vs. F (Fig. 4B; Table 4). An overview of all muscle free amino acid ratios compared to F are shown in the heatmap of Fig. 4D. In summary, after protein supplementation, muscle free BCAA and NEAA were increased with W vs. F, while oral gavage with PS or PS + L did not change BCAA, EAA or NEAA compared to F (Fig. 4D and F).

**Table 3:** End point amino acid plasma concentrations

	F	W	PS	PS + L
Plasma amino acids (µmol/L)				
Isoleucine	85.3 ± 3.6	282.8 ± 10.4^a^	175.5 ± 10.4^ab^	142.9 ± 7.4^ab^
Leucine	150.5 ± 8.4	426.5 ± 15.4^a^	271.0 ± 18.4^ab^	414.4 ± 24.9^ac^
Valine	194.4 ± 9.0	537.5 ± 20.1^a^	383.3 ± 24.0^ab^	331.0 ± 15.1^ab^
**Sum BCAA**	**430 ± 20**	**1247 ± 44** ^**a**^	**830 ± 52** ^**ab**^	**888 ± 46** ^**ab**^
Histidine	68.2 ± 2.5	81.2 ± 3.9	74.8 ± 2.3	70.5 ± 1.5
Lysine	227.1 ± 14.3	566.3 ± 21.6^a^	332.1 ± 19.3^ab^	343.6 ± 14.6^ab^
Methionine	51.8 ± 2.1	117.5 ± 5.7^a^	65.2 ± 3.0^b^	59.6 ± 1.8^b^
Phenylalanine	78.6 ± 3.6	104.2 ± 4.5	115.6 ± 4.7^a^	106.5 ± 3.8^a^
Threonine	163.7 ± 13.1	521.0 21.9^a^	259.2 ± 15.7^ab^	253.7 ± 9.3^ab^
Tryptophan	115.7 ± 10.8	202.7 ± 10.3^a^	147.5 ± 6.4^b^	152.0 ± 8.1^b^
**Sum EAA**	**1135 ± 36**	**2840 ± 82** ^**a**^	**1790 ± 112** ^**ab**^	**1875 ± 70** ^**ab**^
Alanine	329.1 ± 20.0	745.4 ± 44.5^a^	514.9 ± 30.5^ab^	548.4 ± 26.2^ab^
Arginine	39.3 ± 7.8	76.6 ± 9.8	78.8 ± 5.5	71.8 ± 7.2
Asparagine	40.2 ± 4.6	102.2 ± 8.2^a^	87.5 ± 7.1^a^	87.1 ± 4.6^a^
Aspartic acid	20.1 ± 7.0	52.4 ± 9.2	15.6 ± 2.9^b^	20.1 ± 3.5^b^
Citrulline	42.9 ± 2.1	77.1 ± 2.3^a^	65.5 ± 3.3^a^	67.7 ± 2.7^a^
Glutamic acid	73.7 ± 18.8	132.1 ± 12.2^a^	72.6 ± 8.7^b^	85.4 ± 11.3^b^
Glutamine	333.9 ± 33.8	461.8 ± 36.5	354.7 ± 16.1^b^	375.0 ± 12.7
Glycine	201.9 ± 8.2	240.2 ± 11.2	246.8 ± 12.9	250.2 ± 6.2^a^
Serine	113.7 ± 7.3	212.8 ± 13.8^a^	158.4 ± 8.6^b^	162.2 5.0^b^
Tyrosine	95.6 ± 6.6	238.4 ± 14.7^a^	221.3 ± 17.6^a^	200.5 ± 11.2^a^
**Sum NEAA**	**1290 ± 51**	**2339 ± 112** ^**a**^	**1816 ± 83** ^**ab**^	**1868 ± 66** ^**ab**^
**Total AA**	**2529 ± 94**	**5313 ± 177** ^**a**^	**3746 ± 181** ^**ab**^	**3863 ± 130** ^**ab**^

Values are means ± SEM. Significance difference compared to F is shown by ‘a’, compared to W by ‘b’, compared to PS by ‘c’. (One-way ANOVA with SIDAK *post hoc* analysis, *p* < 0.05)

**Table 4:** Muscle free amino acid concentrations

	F	W	PS	PS + L
Muscle free amino acids (µmol/g dry weight)				
Isoleucine	0.31 ± 0.02	0.65 ± 0.02^a^	0.46 ± 0.02^b^	0.33 ± 0.02^b^
Leucine	0.53 ± 0.04	1.02 ± 0.04	0.72 ± 0.03	1.03 ± 0.06^ac^
Valine	0.80 ± 0.04	1.44 ± 0.06^a^	1.13 ± 0.05^b^	0.91 ± 0.04^b^
**Sum BCAA**	**1.6 ± 0.1**	**3.1 ± 0.1** ^**a**^	**2.3 ± 0.1** ^**b**^	**2.4 ± 0.2** ^**b**^
Histidine	0.60 ± 0.02	0.63 ± 0.03	0.63 ± 0.02	0.58 ± 0.02
Lysine	2.77 ± 0.36	4.76 ± 0.35^a^	3.29 ± 0.22^b^	3.20 ± 0.22^b^
Methionine	0.27 ± 0.0	0.46 ± 0.02^a^	0.30 ± 0.01^b^	0.27 ± 0.01^b^
Phenylalanine	0.40 ± 0.01	0.46 ± 0.02	0.50 ± 0.02	0.45 ± 0.02
Threonine	1.20 ± 0.07	2.35 ± 0.13^a^	1.41 ± 0.07^b^	1.33 ± 0.05^b^
Tryptophan	12.67 ± 0.30	12.47 ± 0.37	12.85 ± 0.40	11.91 ± 0.39^a^
**Sum EAA**	**16.8 ± 0.2**	**19.4 ± 0.5**	**18.0 ± 0.5**	**17.0 ± 0.5**
Alanine	12.30 ± 1.83	13.96 ± 1.40	13.62 ± 1.09	13.07 ± 1.28
Arginine	1.09 ± 0.08	1.17 ± 0.07	1.26 ± 0.05	1.20 ± 0.07
Asparagine	0.39 ± 0.07	0.65 ± 0.06^a^	0.58 ± 0.04^a^	0.57 ± 0.04^a^
Aspartic acid	0.60 ± 0.06	1.10 ± 0.09^a^	0.84 ± 0.04^b^	0.94 ± 0.05
Citrulline	0.33 ± 0.02	0.41 ± 0.02	0.39 ± 0.01	0.38 ± 0.02
Glutamic acid	3.10 ± 0.22	5.33 ± 0.25^a^	4.96 ± 0.23^a^	5.25 ± 0.19^a^
Glutamine	6.24 ± 0.31	6.91 ± 0.29	6.21 ± 0.22	6.37 ± 0.22
Glycine	4.36 ± 0.32	4.82 ± 0.23	4.56 ± 0.22	4.43 ± 0.21
Serine	1.19 ± 0.07	1.65 ± 0.23	1.28 ± 0.05	1.27 ± 0.05
Tyrosine	0.48 ± 0.04	0.97 ± 0.06^a^	0.98 ± 0.06^a^	0.86 ± 0.05^a^
**Sum NEAA**	**30.1 ± 1.7**	**38.1 ± 2.5** ^a^	**34.7 ± 1.45**	**34.3 ± 1.5**
**Total AA**	**49.7 ± 1.9**	**63.5 ± 3.5** ^**a**^	**56.2 ± 1.7**	**54.7 ± 2.0**

Values are means ± SEM. Significance difference compared to F is shown by ‘a’, compared to W by ‘b’, compared to PS by ‘c’. (One-way ANOVA with SIDAK *post hoc* analysis, *p* < 0.05)

### Relative bioavailability of leucine

**Fig. 5:**
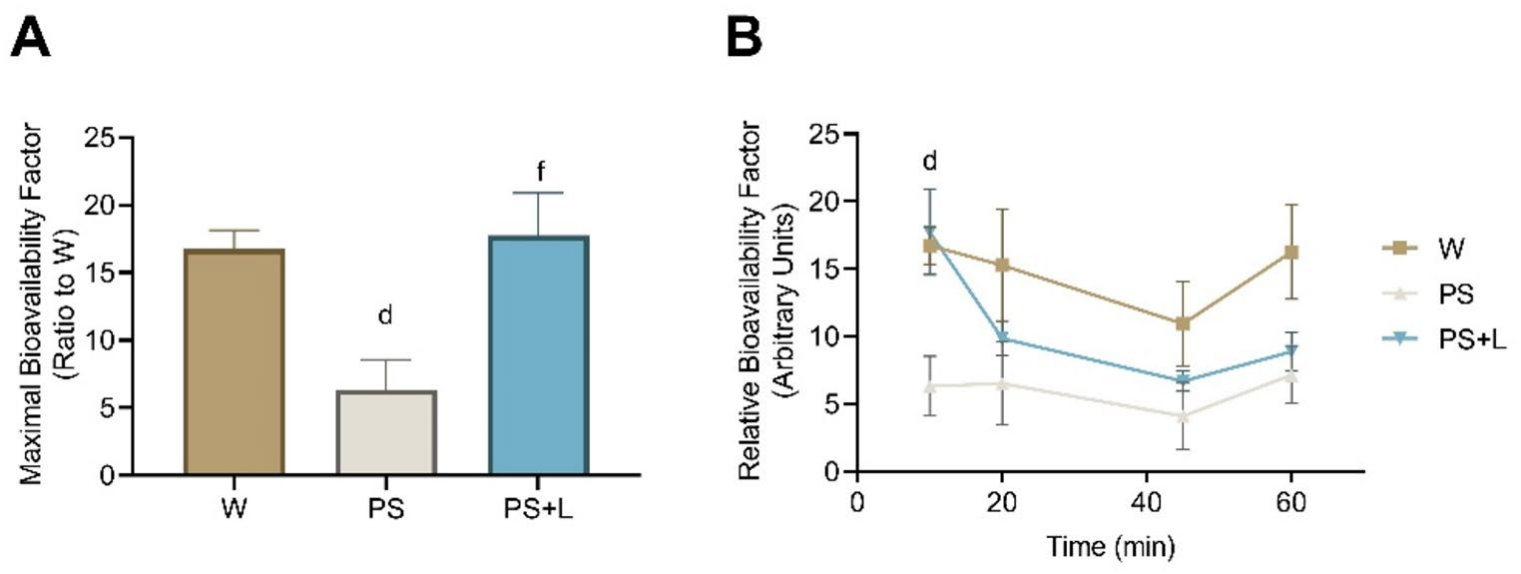
Relative bioavailability factor. **(A)** Maximal bioavailability factor of each group calculated from AUC of DBS leucine data. **(B)** Time curve of relative bioavailability factor of each group calculated from leucine concentrations in DBS. Values are means ± SEM. Significances are shown by *d*: W to PS (One-way ANOVA **(A)** or mixed-effects analysis **(B)** with SIDAK *post hoc* analysis, *p* < 0.05)

Figure 5 shows the relative bioavailability factor as calculated in the Methods section by using the AUC of DBS leucine as depicted in Table 2. Maximal bioavailability factor was reached 10 min post gavage as shown in Fig. 5A. A significant lower maximal bioavailability factor was observed for PS relative to W (*p* = 0.045), whereas PS + L is significantly increased compared to PS (*p* = 0.020) and is comparable to W (*p* = 0.991). The mean relative bioavailability factor at each time point of DBS sampling is shown in Fig. 5B, showing maximal increase 10 min after gavage for W and PS + L (*p* = 0.019 for W; *p* = 0.062 for PS + L compared to PS). However, with PS alone, the bioavailability factor stays low.

## Discussion

This study clearly shows that an optimised blend of pea and soy protein fortified with the anabolic amino acid leucine was equivalent to whey in stimulating MPS in aged mice, whereas a blend of pea and soy alone was not able to stimulate MPS significantly. The effect on MPS stimulation was confirmed by phosphorylation of mTOR signalling pathway protein 4EBP1. Phosphorylated to total ratio of 4EBP1 showed significant activation after W, PS and PS + L supplementations. Although PS supplementation showed a significant increase in phosphorylated to total ratio of 4EBP1, this was not sufficient to increase MPS. The amino acid results suggest that leucine is likely the main driver enhancing the MPS response of the plant-based blend. Other amino acids are lower in PS and PS + L compared to W, so they are less obvious choices to contribute to the MPS response.

Our previous study showed that in this acute setting in aged mice, administration of leucine alone did not increase MPS and building blocks as protein are required [10]. We have previously applied this acute MPS model to test the anabolic potential of a blend of dairy and plant-based proteins (P4) compared to whey, showing that P4 can stimulate MPS to a similar extent as whey in aged mice [26]. The next logical step was to switch to complete PBPs, which we showed in this study with the blend of pea and soy. Since leucine is recognized as a signalling factor for translation initiation and therefore in stimulating MPS [7, 31, 32], we fortified the plant-based blend of pea and soy with leucine. In contrast to our 2018 study [10], we decreased the amount of total protein of the supplement from 139 mg to ~ 70 mg per gavage (0.5 mL). The reason was not to overload the animals with protein supplementation and therefore decrease the sensitivity of the model. Additionally, the fasting period was shortened from an overnight fast [10, 24] to a more physiological morning fast of 6 h [33, 34], which decreases animal discomfort (refinement) and increases sensitivity of the MPS response (derived from previous experiments, data not shown); moreover the adapted model improved translation to the clinical setting. To our knowledge, there were no publications that describe the effect of a blend of pea and soy on acute MPS. Other in vivo studies also compared PBPs, such as wheat and soy, with animal-derived proteins, such as whey and egg, but in a complete meal given for 14 days [9]. The results showed that the Fractional Synthetic Rate (FSR) increased significantly with egg and whey protein but not with wheat or soy protein. Interestingly, Salles et al. [35]. demonstrated that when aged rats were fed 16 weeks a non-isonitrogenous diet with either pea, whey, or casein as protein source, that pea protein was utilized with the same efficiency as casein or whey proteins. No differences were observed in MPS or muscle breakdown. The different outcome of our results compared to the Salles group could be explained by the experimental design; we supplemented the aged mice acutely while Salles provided the diet for 16 weeks and measured basal MPS rates (in 20-months old Wistar rats). Pinckaers et al. [36]. studied the effect of a blend of wheat, corn and pea protein compared to milk protein in healthy young men. In this study, both protein sources contain equal amounts of leucine. The results showed equal increases with both types of supplementations on myofibrillar protein synthesis rates, which could be related to the high amount of leucine in the plant-based blend. In another study Pinckaers et al. [37] compared pea protein to milk protein in young males, showing similar post-prandial muscle protein synthesis rates. This contrasts with our results in aged mice where an additional amount of leucine is needed to obtain an anabolic response with a blend of soy and pea protein, compared to whey protein. Milk protein differs from whey protein as it contains approximately 80% casein and 20% whey, and therefore initiates a different anabolic response to whey. The higher anabolic demand that is required with ageing might be achieved with the additional leucine in our study.

The amino acid profiles of the proteins whey and pea/soy are different (Table 1). Whey contains naturally higher leucine levels, whereas the pea/soy blend is higher in arginine, phenylalanine, and glycine, which all can be reflected in the amino acid plasma levels (Table 3) and muscle free amino acid levels (Table 4). In our previous studies using this model, the limitation was that only the end point blood collection was included (60 min). Now, the repeated blood sampling as DBS provided more insight into the blood amino acids dynamics over time (10, 20, 45 and 60 min). It was interesting to note that within 10 min of ingestion, blood levels significantly increased compared to fasted mice, especially for leucine. This indicates that the protein or free amino acid leucine is processed and transferred through the intestinal system leading to amino acids accessibility in the blood within 10 min. No statistically significant difference was observed in the amount of available plasma leucine between whey (with 7 mg of total leucine), or the PS group with added free leucine (in total 13.2 mg leucine). The leucine present in W is derived from the protein, and a large amount of the leucine in the PS + L group comes from free added leucine. Free leucine can be processed faster and released into the bloodstream, which is what we observed in the DBS data: PS + L has a larger leucine peak at 10 min compared to W or PS.

In this study, methionine and lysine levels are lower in the PS and PS + L blends compared to W. If they are sufficient or not is not known for these aged mice used in this fasting model; however, in the presence of additional leucine, PS + L showed a similar MPS response to W. Both PS supplemented groups showed significantly lower amounts of total EAA and NEAA compared to W. Only PS + L showed an increased MPS response. This suggest that sufficient building blocks were provided, but the addition of leucine is required to initiate the MPS response.

Bioavailability of amino acids is associated with better functionality and quality of the protein. As such, we wanted to explore the relative impact of leucine from intact protein as well as the potency of the free leucine on increasing the level of amino acid in the blood. Several methods to calculate bioavailability or digestibility of a (protein) meal can be found in literature (reviewed by [38, 39]). However, many methods require techniques that we did not use in this study. Therefore, we used an alternative method to calculate the relative bioavailability by using the DBS data and the amount of leucine that was supplemented by oral gavage. Hence, we can quantify and compare the relative potency of different amino acids from different sources. The results obtained from the AUC of the DBS data showed that PS alone had a significantly lower relative bioavailability factor compared to W, while the relative bioavailability factor of PS + L was higher than PS only (no significant difference between PS + L and W). In time, there was a clear difference in relative bioavailability factor between W, PS + L and PS alone, which was most significant 10 min after gavage. As also concluded from the DBS data itself, with this relative bioavailability factor it is confirmed that the fast increase is due to the free leucine in PS + L, sufficient to initiate the MPS response in this experimental setup.

There are certain limitations to this study which should be addressed. This study describes the acute effect of a single bolus of protein in aged mice to measure MPS, which does not represent the situation of a complete meal where also other macronutrients such as fats and carbohydrates are present. MPS response with added carbohydrates and fats of a complete meal might alter the MPS response of the proteins. Another limitation of our study is that we did not measure other aspects of protein metabolism, such as protein breakdown, and we cannot discriminate between the free added leucine and the leucine incorporated in the protein sources. It would be interesting to understand how much of the free leucine is utilized for MPS. Finally, the use of additional isonitrogenous control groups, e.g. single pea or soy, could further substantiate the importance of the effect of blending PBP sources, although previous studies clearly show that single PBP sources are inferior compared to whey in generating a MPS response in aged individuals [12, 15, 19].

A strength of this study is that we demonstrated once more the use of the SUnSET method [28] to measure MPS in vivo in both fasted and postprandial conditions, which was reinforced by the confirmation of the mTOR pathway signalling protein 4EBP1. Moreover, this study showed that the implementation of the DBS provides more insight into the dynamics of amino acid availability during the postprandial period. The end point amino acid values were represented by the DBS results, if results are accepted to be relative instead of absolute quantities. The DBS make it possible to reduce number of animals and animal discomfort, and can be implemented in different experimental setups, not only in vivo animal studies, but also clinical human studies, especially with very young or frail participants.

The clinical relevance of our data lies in the nutritional support for leucine-enriched plant-based alternatives to whey effectively stimulating MPS in older individuals. Extrapolating this to the human situation (e.g. 75 kg male), the ~ 70 mg provided in this study equals 12.2 g protein in humans. For total leucine in the PS + L blend, ∼13.2 mg provided in aged mice represents 2.3 g leucine in humans [40]. These are suboptimal amounts compared to the study where a leucine-enriched whey protein supplement (20 g whey protein, 3 g total leucine) effectively increased MPS in healthy older adults [41] and increased muscle mass after 3 months of intervention [8]. The PRO-TAGE study group also published a position paper, recommending the intake of 2.5–2.8 g leucine per meal for older adults [42]. It would be very worthwhile to repeat these interventions in the older adults.

In conclusion, a blend of pea and soy, fortified with leucine can stimulate MPS to a similar extent as whey protein in this acute murine ageing model. Whilst other differences in plasma and muscle amino acid levels were evident, leucine appears to be the main driver for the anabolic responses observed. These findings may be relevant for application within the frail and ageing population and could provide an alternative to current animal-derived protein sources with similar benefits on muscle protein synthesis.

## Data Availability

Raw data is available upon request.
